# P01-015 – Effect of Cochicine on cholesterol in FMF and BS

**DOI:** 10.1186/1546-0096-11-S1-A19

**Published:** 2013-11-08

**Authors:** S Ugurlu, E Seyahi, I Hanci, SM Pehlevan, H Ozdogan, H Yazici

**Affiliations:** 1 Department of Internal Medicine, Division of Rheumatology, Cerrahpasa Medical Faculty, University of Istanbul, Turkey; 2 Department of Internal Medicine, Division of Rheumatology, University of Fatih, Istanbul, Turkey

## Introduction

We and others have previously shown that patients with Familial Mediterranean Fever (FMF) had low cholesterol levels when compared to healthy controls [1,2]. This was initially brought up by Ozkan E [3]. The causes of this abnormality are not understood. It could be due to an inherent effect of FMF or due to a lipid lowering effect of colchicine, as the patients in these studies were all regular users. Additionally, earlier studies had suggested that colchicine may have hypocholesterolemic effect.

## Objectives

We conducted a 12 week study to determine whether colchicine would decrease serum lipid levels in patients with FMF and Behçet's syndrome (BS). Lipid levels were measured in each patient before and after colchicine use.

## Methods

Blood cholesterol and triglycerides levels were measured in 24 patients with FMF (11 M, 13 F) and 16 (8 M, 8 F) patients with BS who were registered at the outpatient clinic of Cerrahpasa Medical Faculty. All patients were naive to colchicine or immunosuppressive treatment or any other lipid lowering drugs at study entry. Blood cholesterol and triglycerides levels were measured again after 12 weeks of colchicine 1.5 mg daily. Colchicine was withdrawn in one patient with FMF because of liver toxicity and in another because of nausea. Two patients with FMF did not use colchicine and another with FMF was lost to follow-up. Colchicine was switched to azathioprine in 1 patient with BS because of active disease. Only patients who completed 12 weeks period were analyzed.

## Results

There were 19 (8 M, 11 F) patients with FMF and 15 (7 M, 8 F) patients with BS who completed the 12 week period. Patients with FMF were (mean age: 33.8±14.1 years) significantly younger than BS patients (mean age: 36.5±9.5) (P = 0.001). Colchicine did not change cholesterol and triglycerides levels in patients with FMF (T.Cholesterol: 169±77 vs 181±48 mg/dl, P = 0.58, Triglycerides: 122±82 vs128±70 mg/dl, P= 0.75, LDL:120±44 vs 112±40, P=0.35, HDL: 42±13 vs 47±11 mg/dl, P=0.1, before and after colchicine use, respectively). This was also true for BS patients (T.cholesterol:181±51 vs 172±44 mg/dl, P=0.53, triglycerides:112±63 vs 107±52 mg/dl, P=0.18, LDL:115±38 vs 106±40 mg/dl, P = 0.85, HDL:48±9 vs 48.3±9.9 mg/dl, P= 0.3).

## Conclusion

This study provided no evidence that colchicine use affects lipid levels in patients with FMF and BS.

## Disclosure of interest

None declared.
